# Protein structure determination via an efficient geometric build-up algorithm

**DOI:** 10.1186/1472-6807-10-S1-S7

**Published:** 2010-05-17

**Authors:** Robert T Davis, Claus Ernst, Di Wu

**Affiliations:** 1 Department of Mathematics and Computer Science, Bioinformatics and Information Sciences Center, Western Kentucky University, Bowling Green, KY 42103, USA

## Abstract

**Background:**

A protein structure can be determined by solving a so-called distance geometry problem whenever a set of inter-atomic distances is available and sufficient. However, the problem is intractable in general and has proved to be a NP hard problem. An updated geometric build-up algorithm (UGB) has been developed recently that controls numerical errors and is efficient in protein structure determination for cases where only sparse exact distance data is available. In this paper, the UGB method has been improved and revised with aims at solving distance geometry problems more efficiently and effectively.

**Methods:**

An efficient algorithm (called the revised updated geometric build-up algorithm (RUGB)) to build up a protein structure from atomic distance data is presented and provides an effective way of determining a protein structure with sparse exact distance data. In the algorithm, the condition to determine an unpositioned atom iteratively is relaxed (when compared with the UGB algorithm) and data structure techniques are used to make the algorithm more efficient and effective. The algorithm is tested on a set of proteins selected randomly from the Protein Structure Database-PDB.

**Results:**

We test a set of proteins selected randomly from the Protein Structure Database-PDB. We show that the numerical errors produced by the new RUGB algorithm are smaller when compared with the errors of the UGB algorithm and that the novel RUGB algorithm has a significantly smaller runtime than the UGB algorithm.

**Conclusions:**

The RUGB algorithm relaxes the condition for updating and incorporates the data structure for accessing neighbours of an atom. The revisions result in an improvement over the UGB algorithm in two important areas: a reduction on the overall runtime and decrease of the numeric error.

## Introduction

Proteins are important bio-molecules in biological systems and activities. A protein is a polypeptide chain made of 20 different types of amino acids. An amino acid sequence determines the structure of the protein. Knowledge of the protein structure gives us insight into function of the protein and its dynamics. Therefore, it is always important to have an accurate protein structure in the highest resolution available. The distances between many pairs of atoms in a protein can often be determined based on our knowledge of chemistry (for example certain types of bond-lengths and bond angles) [1], or from nuclear magnetic resonance (NMR) experiments [2]. If a sufficiently large set of inter-atomic distances can be obtained, then a protein structure can be determined by solving a so-called molecular distance geometry problem (MDGP) [3]. MDGPs in their most general form are known to be computationally intractable (NP-hard) [4].

In an experimental setting there are two additional restrictions: First, often only a small subset of all pair-wise distances may be available. Second, instead of a single distance, experiments might only yield a distance range for a pair of atoms (a lower bound and upper bound of a distance). Several algorithms have been developed as solutions or approximate solutions to MDGP. These algorithms include singular value decomposition [3], the embedding algorithm [3], the alternative project algorithm [5], the graph reduction algorithm [6], the multi-scaling algorithm [7], and the global optimization algorithm [8][9]. Many of these algorithms are computationally expensive, in particular if they attempt to solve the MDGP in a general form.

In this paper we will only consider the MDGP in the case when exact distances are available. Furthermore we concentrate on a particular class of algorithms that are computationally quite fast and will often suffice to solve the MDGP problem. These are so called geometric build-up algorithms (GB) [10]. A GB algorithm is based on the idea of iteratively adding one atom at a time to a list of positioned atoms.

Here we will refer to a positioned atom as an atom with known coordinates in 3D space and an unpositioned atom as an atom where we do not know its 3D coordinates. It is well-known in geometry that in 3D an unpositioned point *P* can be positioned when there exist four positioned non-planar points, each of which has a known distance to *P*. It is easy to see that when all pair-wise distances between atoms are available, a set of four such atoms can always be found. Such a set of four atoms used to determine another atom is also called a *metric base*. The algorithm in the case when all distances are known has a linear running time because a metric base is easily found [10]. However, such an ideal situation will hardly ever arise. Clearly in this case the MDGP is not a hard problem. The algorithm needs to be modified to determine a protein structure when only a sparse set of pair-wise distances is available. In such a case, finding a metric base to add an atom to the list of positioned atoms requires more work. The simplest idea would be to exhaustively search through all possible metric bases until one is found that allows the positioning of an atom. Theoretically, when a sparse distance data has sufficiently many entries a protein structure can be determined.

A major problem in the geometric build up procedure is numerical stability when a protein has a large number of atoms. Due to computational round off or truncation, errors are introduced into the build-up coordinates and the iterative nature of the algorithm can cause these errors to accumulate. This problem has been solved by using an updated geometric build-up (UGB) algorithm [11]. The updating reduces the accumulation of numerical error to a tolerable level. The UGB algorithm can solve the MDGP with high accuracy. The idea of the updating procedure is to re-compute the coordinates of the four atoms in the metric base whenever possible using the original, correct pair-wise distances. Therefore, the fresh coordinates of these four atoms, with a minimal numerical error, can be used to determine the unpositioned atom more accurately. The drawback of the UGB algorithm is the additional computational time it requires to select a metric base carefully and the updating procedure itself.

### Geometric build-up algorithms

**Figure 1:**
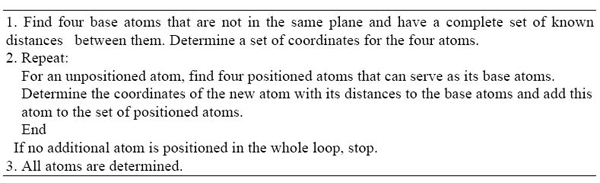
The outline of the General Geometric Build-Up Algorithm for Solving MDGP [Dong and Wu 2002b]

Initially, four atoms that are not co-planar are selected such that all six inter-atomic distances between each pair of these four atoms are known. A set of coordinates for the four atoms is determined that satisfies the distances between them. We call atoms with fixed coordinates *positioned* atoms. Thus there are initially only four positioned atoms. Next, the GB algorithm increases the number of positioned atoms by determining the coordinates of an unpositioned atom that has four known distances to four distinct non-planar already positioned atoms. As before, we call these four already positioned atoms *a metric base* of the unpositioned atom. Using this procedure repeatedly all the coordinates of the remaining atoms can be determined using a distance from four positioned atoms. The algorithm can solve a MDGP even when only a sparse set of pair wise distances are available. In this case, the metric base of four base atoms may need to be changed frequently in the determination of the remaining atoms. This general geometric build-up algorithm is outlined in the Figure 1 and also in reference [12]. The geometric build-up (GB) algorithm will solve a MDGP when a sufficiently large set of exact pair wise distances is given. It is possible to formulate conditions that grantee a sufficiently large set of distances so that a geometric build-up algorithm will be successful, for details see [13].

**Definition 1.1** A set of points *B* (with known coordinates) in a space (usually *R^3^*) is a *metric basis* of a set *S* of points provided the coordinates of each point of *S* are uniquely determined by its known distances to the points in *B*.

**Definition 1.2** A set of four points in *R^3^* is called *independent* if they are not co-planar.

**Definition 1.3** A point *u_i_* is called a *neighbouring point* of a point *u_j_* if *u_i_* has a known distance *d_i,j_* from *u_j_*.

**Theorem 1.1** Given a set of distances among four non-coplanar points, then the coordinates of the four points can be uniquely determined up to a rigid motion that is a combination of a translation, a rotation and possibly a reflection.

**Proof.** The distances between the four points define a tetrahedron and therefore this is obvious.

**Theorem 1.2** If the coordinates of four non-planar atoms *x_i_, i=1,2,3,4* and the distances *d_i,j,_ , i=1,2,3,4* to a fifth atom *x_j_* are given, then the coordinates of the fifth atom *x_j_* can be determined uniquely. In other words, any four independent points in *R^3^* form a metric basis for *R^3^*.

**Proof.** While this theorem is geometrically obvious, we provide a short proof that will give us insight of how the coordinates of the fifth atom are actually computed. Let *x_i_ =* (*u_i_*,* v_i_*,* w_i_*)*^T^*, *i* = 1, 2, 3, 4, be the coordinate vectors of the first four atoms and *x_j_ =* (*u_j_*,* v_j_*,* w_j_*)*^T^* the coordinate vector of the fifth atom. We then have a set of equations,

Square the equations and expand their left-hand-sides to obtain

Subtract the first equation from the rest to reduce the equations to the following three,

Define the matrix *A* and the vector *b* by:

We can then write the above equations in the following matrix form.

Since *x*_1_, *x*_2_, *x*_3_, *x*_4_ are not in the same plane, the matrix *A* is nonsingular and therefore, the linear system of equations can be solved to obtain a unique solution for *x_j_*. Therefore, any four independent points in *R^3^* form a metric basis for *R^3^*.

Note that the above algorithm (given in the proof of Theorem 1.2) shows that the coordinates of *x_j_* can be computed in a constant time. Therefore, the geometric build-up algorithm can determine a protein structure in a linear running time when all exact distances are available. Moreover, when all distances are available a single metric base can be used throughout the process because in each iteration as the four required distances will be available. However there is no guarantee that a solution to the MDGP can be found when only sparse distance data is available. If we assume that any initial four atoms will lead to a protein structure using the GB, the algorithm will require a *O(n^3^)* running time in a worst case analysis. There are three nested loops in the GB algorithm: A while-loop (while L is not empty, where L is the list of unpositioned atoms), within the while-loop a for-loop (check all remaining atoms in L to find which one can be determined with currently determined atoms), and within the for-loop finding four determined atoms with a distance from a given atom. Each step has in the worst case *O(n)* many steps. Therefore, the worst case total running time is* O(n^3^)*.

As shown in previous reports [12], sparse distance data can produce a large numerical rounding error that must be dealt with. In the case of given sparse distance data, almost always a new different metric base must be used in the determination of a single atom. Thus, the metric bases used in determination of unpositioned atoms contain rounding errors from earlier calculations. Therefore the errors introduced in previous steps accumulate. As a result, the matrix A in the proof of Theorem 1.2 is often not accurate and hence cannot be used to determine new coordinates of atoms accurately. In summary, the GB algorithm produces larger and larger rounding error in the coordinate determination of unpositioned atoms.

### An updated geometric build-up algorithm (UGB)

This algorithm incorporates the idea of re-computing the coordinates of the four atoms in a metric base to minimize the rounding error. In many cases, there exist many options to select a metric base of four atoms that can determine an unpositioned atom. In the updated geometric build-up algorithm, four non-coplanar atoms with original distances among them are preferred. The reason is that a metric base forms a tetrahedron *T* consisting of original distances that allows to position the atoms of the metric base relative to each other with minimal rounding error. The coordinates of the unpositioned atom can now be determined with minimal rounding error relative to the tetrahedron *T* creating a complex consisting of 5 atoms whose edges form a complete graph *K_5_*.

**Figure 2:**
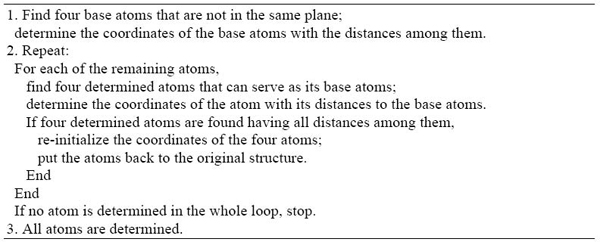
The outline of the updated geometric build-up algorithm for solving the molecular distance geometry problem with sparse exact distances [Wu and Wu]

It is important to realize that the determination of the coordinates of the unpositioned atom is independent of the coordinates of other atoms obtained previously. After translation and rotation of the complete graph *K_5_* consisting of the five atoms (the metric base and unpositioned atom) will be put back into the protein structure in a way that will minimize the rounding errors of the positions of all 5 atoms simultaneously. The old already build-up coordinates of the four atoms in T (with their potential error) will be replaced by the new updated set. We call this procedure re-initializing the coordinates of the five atoms. This algorithm is outlined in the Figure 2 and also in reference [11].

There are two major steps in this algorithm. First, the positions of the four base atoms are recomputed based on Theorem 1.1. The new positions of the four base atoms are completely independent of their old positions, and this first step just guarantees that the four base atoms form a tetrahedron where the distances between the atoms as accurate as possible. Second, the translation vector and rotation matrix need to be found for re-initializing. This second step requires techniques used in computation of the Root Mean Square Deviation (RMSD).

We explain the re-initialization step for a tetrahedron when all distances among four atoms are available. Let (*x_i_, y_i_, z_i_*) be coordinates of *i ^th^* atom, *i=1,2,3,4,* four atoms and let *d_ij_* be the distance between *i ^th^* and *j ^th^* atoms, *i=1,2,3,4.* The initialization consists of the following steps. We put the first atom at the origin, the second atom on the *x*-axis and the third atom into the *xy*-plane. Then we can determine the position of the fourth atom. The formulas below explain the above steps and a more detailed explanation of the procedure is available in the reference [11],

x_1_=0, y_1_=0, z_1_=0

x_2_=d_21_, y_2_=0, z_2_=0

We explain the standard RMSD steps for any two structures of embedded points with coordinate matrices *X* and *Y* of an identical set of *n* points. In our case *n=4*, the matrix *X* contains the old coordinates of metric base atoms and the matrix *Y* contains the recomputed coordinates of the metric base atoms. First, we need to translate these two structures so that their geometric centers are both at the origin. This can be done using the following formulas,

,  for *j* =1,2,3.

 for *i* =1,2,…,n.

Now, *X_1_* and *Y_1_* are the two translated  matrices with the same geometric center at the origin. We can then find the rotation matrix *Q* so that RMSD value of *X_1_* and *Y_1_* is minimized. This is formulated as , where *Q* is a rotation matrix and || ||_F_ is defined by , where is the distance between the two points *X_i_* and *Y_i_*. *Q* can be computed through the following steps. Compute *C= Y_1_^T^X_1_*; then let *UΣV^T^*=C be the singular value decomposition of *C.* That *Q=UV^T^* can be easily verified to be the solution to the above minimization problem. In the updated geometric build-up algorithm, the above computations will give the translation vectors *(xc(1),xc(2),xc(3))* and *(yc(1),yc(2),yc(3))* and the rotation matrix *Q*. Applying this to the recomputed coordinates of four metric base atoms and the newly determined atom, the five atoms can be translated and rotated back to the protein structure. Compared to the general geometric build-up algorithm, in many cases, only the updated geometric build-up algorithm can determine protein structures completely and accurately when a sparse set of distance data is available [9]. However, the algorithm has a drawback that a brute force search for a metric base of four atoms with known distances among them can take up to *O(n^4^)* (if one considers all 4 element subsets of *n* points) and then the total running time can be *O(n^6^)*. The majority of this worst case running time is spent finding four atoms with all distances among them.

In this paper, the UGB algorithm is improved by a revised updated geometric build-up algorithm (RUGB). This algorithm aims at reducing the computational complexity of the UGB algorithm. As we will show the RUGB algorithm also improves the numerical error over the performance of the UGB algorithm.

## Methods

### A revised updated geometric build-up algorithm (RUGB)

Although the updated geometric build-up algorithm UGB has shown the property of controlling numerical errors, the UGB algorithm requires searching for four atoms with distances among them as a metric base in every iteration. A revised updated geometric build-up algorithm is described in this paper. The algorithm is based on the regular updated geometric build-up algorithm and modified by adding a new data structure and relaxing the condition of a metric base. The first modification in the algorithm is that instead of requiring four metric base atoms with distances among them, this algorithm requires three metric base atoms with distances among them and one additional atom. The purpose of relaxing the condition is to cut down the time it takes to find a new metric base. The updating scheme can still be implemented with only three metric base atoms. However, using three atoms, with all distances among them, will result in two possible sets of coordinates for the position of an undetermined atom. In order to distinguish the correct solution from the incorrect solution we use the distance to a fourth determined atom that is not in coplanar with the first three base atoms. This strategy is also based on Theorem 1.2. The re-initialization and updating of the metric base of three atoms also follows the steps similar to those in UGB algorithm introduced in the previous section. In this case, three atoms rather than four atoms are considered.

**Figure 3:**
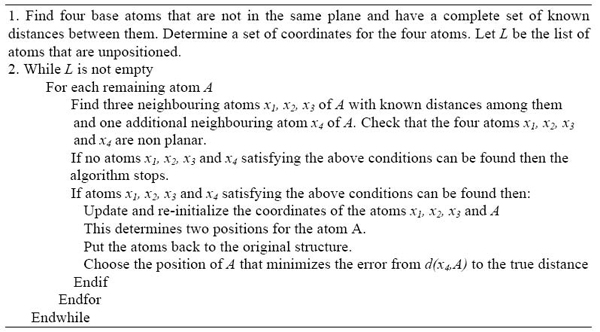
The outline of the revised updated geometric build-up algorithm (RUGB) for solving the molecular distance geometry problem with sparse exact distances

A second modification is the creation of a data structure that makes it easy to access all of the neighbouring atoms given by the original distance matrix for any atom. Here we refer to the degree of an atom as the number of neighbouring atoms and *d_max_* as the largest degree of all the atoms. Using the original distances we generate a list of adjacency arrays, whose lengths are bounded by *d_max_*. Then searching through these lists of neighbouring atoms, three metric base atoms and one additional atom can be much faster because *d_max_* is typically small when compared to *n* the number of all atoms, especially as *n* gets large. Recall that previously the UGB algorithm may require in the worst case an exhaustive search through all subsets of four atoms out of *n* atoms. The relative size difference between the number of atoms *n* and *d_max_* is illustrated in Table 1, consisting of the ten largest of the tested proteins. Note that in Table 1 the size of *d_max_* is somewhat dependent on how the data set is generated. However we assume that this is still typical for a sparse data set. The revised geometric build-up algorithm UGB is outlined in Figure 3. Two theorems illustrate the computational complexity of the revised geometric build-up algorithm.

**Table 1:** The size of *d_max_* compared to the total number of atoms *n*

PDB Name	# Atoms	***d_max_***	***d_max_/n***
'1VII.pdb'	596	77	0.129195
'1HIP.pdb'	617	37	0.059968
'1ULR.pdb'	677	36	0.053176
'1BOM.pdb'	700	69	0.098571
'1AIK.pdb'	729	49	0.067215
'1CEU.pdb'	854	65	0.076112
'1KVX.pdb'	954	38	0.039832
'1VMP.pdb'	1166	74	0.063465
'1HSM.pdb'	1251	73	0.058353
'1HAA.pdb'	1310	69	0.052672

**Theorem 3.1** Assume that any four initial metric base atoms can lead to the complete determination of a protein structure given a sparse set of distance data, then a protein structure can be determined by the revised geometric build-up algorithm (RUGB) using *O(n^2^d_max_^3^)* many steps, where *n* is the number of atoms and *d_max_* is the largest degree of atoms

**Proof.** For any unpositioned atom *A*, it will take *O(d_max_^3^)* many steps to know if there exist three neighbouring atoms *x_1_, x_2_, x_3_*, which have known distances between them. If it is the case, then it will take *O(d_max_)* many steps to know if there is any additional neighbouring atom *x_4_* of *A* such that *x_1_, x_2_, x_3_*, and *x_4_* are non planar.

If both a metric base of three atoms *x_1_, x_2_, x_3_* and an additional neighbouring atom *x_4_* can be found, then apply the updating strategy, which includes re-computing the coordinates of a metric base of three atoms *x_1_, x_2_, x_3_*, determining the coordinates of the unpositioned atom *A*, updating coordinates by translation and rotation and using the additional atom *x_4_* to determine the correct position for *A*. For any choice of the four atoms *x_1_, x_2_, x_3_*, and *x_4_* this can be done in constant time.

Thus for an unpositioned atom *A* the total running time will be at most *O(d_max_^3^)* regardless if the position of *A* can be determined at this point. There are at most *n* unpositioned atoms and in the worst case we have to look at all of them before we can add a single atom. Thus it may take *O(nd_max_^3^)* many steps to add a single atom. Since the size of the initial list L is *n-4* initially, the total running time is *O(n^2^d_max_^3^)*.

Note that often in NMR structure determination, only distances less than 5Å can be obtained. Therefore, the typical distance matrix is sparse in realistic applications. However, the RUGB algorithm of Figure 3 relies on the successful selection of the initial four metric base atoms. There is no guarantee that choosing any arbitrarily selected metric base for initialization, will result in the algorithm completely determining a protein structure. In such a case we can start over by selecting a different set of atoms for initialization. The following theorem analyzes the upper bound of computational complexity no matter whether a protein structure can be determined or a graph can be realized, using a revised geometric build-up algorithm.

**Theorem 3.2** Given a sparse set of distance data for a protein, then it takes at most *O(n^3^d_max_^6^)* to determine whether a protein structure can be solved using a revised geometric build-up algorithm.

**Proof.** In a protein structure, there are at most *O(nd_max_^3^)* many four atoms that are non co-planar and have distances among them. Any of these sets of four atoms can be considered an initial metric base. However, the worst case is all of them fail until the last one works or none of them work at all. Therefore, the upper bound of running time is *O(nd_max_^3^) O(n^2^d_max_^3^)*=* O(n^3^d_max_^6^)*.

## Results

We tested the RUGB algorithm on a set of proteins. We also compared the results with results generated by the GB algorithm and the UGB algorithm. The testing data was prepared in the following way. A set of proteins with their structures were downloaded from the protein structure database PDB [14]. For each protein, a structure file contains the *(x,y,z)* coordinates corresponding to each atom in the structure and then a distance matrix of all pair wise distances can be generated. In practice, especially in NMR experiments, only distances between two protons less than 5Å are typically available. In our testing we used a cut-off distance of 5 Å and deleted all distances that were larger (if there were any). This resulted in sparse distance data that only contains distances less than 5Å. However, due to the poor performance of general GB algorithm on sparse distance data, we also generated a second matrix using a different cut-off distance of 8Å. For each test case of a protein, we applied the GB, the UGB and the RUGB algorithms. We analyzed results by comparing numerical error and running time for the three algorithms.

The Table 2 lists numerical results of a set of proteins tested on the RUGB algorithm and a regular UGB algorithm. The first column contains PDB IDs of tested proteins; the second column contains the number of atoms in each protein; the third column shows the running time using RUGB (in seconds); the fourth column shows the running time of using UGB (in seconds); the fifth column shows the RUGB RMSD error between the determined structure and the real structure; the six column shows the UGB RMSD error between the determined structure the real structure.

**Table 2:** The numerical results of RUGB and UGB

PDB Name	# Atoms	RUGB time (s)	UGB time (s)	RUGB error (Å)	UGB error (Å)
'2DX2.pdb'	174	3.5803	4.2447	2.31E-11	1.71E-08
'1ID7.pdb'	189	3.0529	4.4187	8.62E-14	2.87E-12
'1B5N.pdb'	332	8.1185	10.1274	1.93E-10	8.67E-08
'1FW5.pdb'	332	6.9327	9.6053	1.65E-12	6.29E-08
'1SOL.pdb'	353	8.318	13.5202	7.33E-13	5.72E-11
'1JAV.pdb'	360	7.9572	11.4536	2.78E-12	1.50E-08
'1meq.pdb'	405	8.7641	14.076	2.43E-12	1.20E-10
'1AMB.pdb'	438	13.966	16.9998	7.11E-12	4.35E-07
'1R7C.pdb'	532	13.3252	26.2002	8.62E-10	5.50E-08
'1HLL.pdb'	540	13.0888	28.5319	2.83E-12	5.41E-07
'1VII.pdb'	596	13.0338	24.7907	3.56E-10	2.28E-07
'1HIP.pdb'	617	15.9565	35.5588	4.80E-10	5.45E-07
'1ULR.pdb'	677	19.9154	127.6762	3.84E-10	5.43E-11
'1BOM.pdb'	700	15.6276	37.5214	1.36E-09	3.16E-09
'1AIK.pdb'	729	17.302	39.4843	9.19E-09	7.89E-09
'1CEU.pdb'	854	21.3126	49.3975	3.15E-10	2.43E-09
'1KVX.pdb'	954	27.6469	83.2725	7.21E-04	6.61E-04
'1VMP.pdb'	1166	32.7741	95.3844	1.01E-06	5.57E-06
'1HSM.pdb'	1251	37.8582	108.2448	5.88E-07	3.22E-07
'1HAA.pdb'	1310	35.6037	129.6353	4.49E-10	8.25E-07

E-5 means 10^-5^ and E+5 means 10^5^; others follow similarly

**Figure 4:**
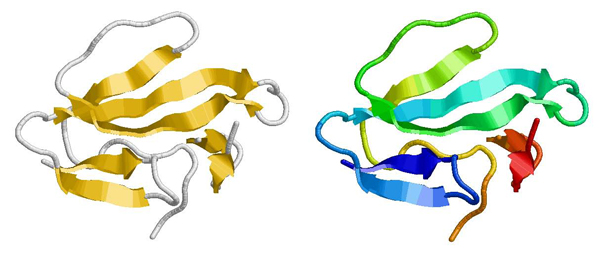
**Protein 3D structure determined by RUGB and the original protein 3Dstructure of 1HAA.** The left picture is for protein 3D structure of 1HAA determined by RUGB and the right picture is for protein the original protein 3Dstructure of 1HAA

In Table 2 the RUGB algorithm shows a decreased runtime in all of tested proteins when compared to the regular UGB algorithm. In particular, for some large proteins such as 1HSM and 1HAA having 1251 and 1310 atoms respectively, the RUGB algorithm can determine each protein structure about 3 times faster. In addtion, the RUGB algorithm results in less RMSD error for most proteins when compared with the UGB algorithm. Figure 4 shows that our determined protein structure of 1HAA (on the left) is practically identical to the original structure of 1HAA deposited in PDB (on the right). Both structures are displayed in Rasmol [15].

This is surprising since the up-date regimes are very similar. The main reason could be the following: The RUGB algorithm uses only three base atoms to numerically determine an unpositioned atom with two solutions and one additional atom to fix the real solution. This up-dating procedure involves less numerical calculation when compared with the 4 atom up-dating routine of the UGB algorithm. So it could be that the RUGB up-dating produces a smaller numerical error.

**Figure 5:**
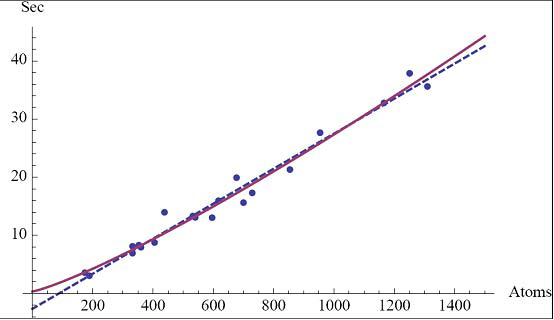
Plot of run-time of the UGB algorithm with the two best-fit functions

The theoretical analysis (Theorems 3.1 and 3.2) discuss the upper bound of run-time of RUGB. Clearly the numerical data shows that the algorithm runs much faster than the theoretical worst-case analysis using the proteins in our data set. The run-time data is plotted in Figure 5. Since all proteins selected in our example can be determined with any initial four atoms, our results should show a much better run-time than Theorem 3.2. However, the numerical results also show a runtime that is better than Theorem 3.1. Recall that the theoretical result in Theorem 3.1 only shows an upper bound on the run-time of the RUGB algorithm. In addition, in the way we constructed our data sets the algorithm may not require *O(n)* steps in the while-loop to find an unpositioned atom whose coordinates can be determined. Therefore, the results show a lower than quadratic runtime behavior in our tests. Our data compares nicely with the linear fit,  (see dashed line in Figure 5). However, the non-linear fit  (see solid line in Figure 5) produces a slightly higher correlation coefficient. (A log-log computation using least square shows that *r^2^* is maximal for the power *n^1.2^*.)

In Table 2, the structural determination of 1KVX shows unusually larger numerical errors, compared with several other selected proteins that have a similar number of amino acids, such as 1CEU and 1VMP. One reason might be that a triangle selected in the RUGB algorithm leads to a very flat tetrahedron. In this case the positions of four atoms are almost co-planar, and the determination of position of the unknown atom produces a solution of coordinates with a larger error then the error produced by a tetrahedron that is not consisting of four almost coplanar points.

The Table 3 compares the results of the RUGB algorithm and the regular GB algorithm for the same proteins as Table 2. The first three columns are identical to the corresponding columns in Table 2. The fourth column shows the RMSD error between the structure determined by GB and the real structure with the distance matrix using the 8Å cut-off distance; the fifth column shows the same as the fourth column but using a distance matrix with a 5Å cut-off distance.

**Table 3:** Numerical results of using RUGB and GB methods in protein structure determination

PDB Name	Atoms	RUGB error*	GB error1**	GB error2*
2DX2	174	2.31E-11	7.81E-12	4.80E-05
1ID7	189	8.62E-14	1.94E-13	8.48E-08
1B5N	332	1.93E-10	1.87E-07	4.31E+07
1FW5	332	1.65E-12	2.31E-08	1.55E+00
1SOL	353	7.33E-13	1.58E-05	1.70E+04
1JAV	360	2.78E-12	3.33E-03	4.97E+01
1MEQ	405	2.43E-12	4.54E-08	2.21E+04
1AMB	438	7.11E-12	3.01E-09	1.11E+00
1R7C	532	8.62E-10	1.54E-2	6.07E+12
1HLL	540	2.83E-12	2.04	1.83E+09
1VII	596	3.56E-10	0.373	1.52E+05
1HIP	617	4.80E-10	1.25E+5	N.A.
1ULR	677	3.84E-10	3.20E+3	7.33E+09
1BOM	700	1.36E-09	2.7E-2	1.68E+12
1AIK	729	9.19E-09	26.9	N.A.
1CEU	854	3.15E-10	5E-5	9.35E+09
1KVX'	954	7.21E-04	977.49	7.45E+30
1VMP	1166	1.01E-06	2.78071E+13	N.A.
1HSM	1251	5.88E-07	1857.809626	1.37E+15
1HAA	1310	4.49E-10	83.15	6.62E+09

All errors are in Å.

N.A. means such protein can not be determined due to a large numerical error

E-5 means 10^-5^ and E+5 means 10^5^; others follow similarly

* for each tested protein, a given set of distances are prepared with a cut-off distance 5Å

** for each tested protein, a given set of distances are prepared with a cut-off distance 8Å

In Table 3, it is easy to see that the updating procedure plays a very important role in controlling numerical errors, see also similar results in [9]. Using a 8Å cut-off distance, the GB algorithm can determine the structure all tested proteins in some sense, however the rounding errors are so large that these structures are no longer useful.

Using a 5Å cut-off distance, the GB algorithm fails in producing a complete protein structure in some instances due to a round-off error that gets out of control. For the 8Å cut-off distance the given set of pair wise distances is much denser. This work verifies that the importance of updating that is used in both the RUGB and the UGB algorithms. Both algorithms indeed can determine a protein structure with a high accuracy.

## Conclusions

A very accurate protein structure is essential to understand the function and dynamics of the protein in biological systems and activities. Applications of distance geometry in protein structures determination arise from the fact that pair wise distances of atoms in a protein can often be obtained from experiments or our knowledge of chemistry. Hence a protein structure can be determined if there exists a solution to the distance geometry problem. However, the problem is proved to be NP-complete. GB algorithms do not solve all distance geometry problems. In the cases where they do give a solution, GB algorithms can determine protein structure efficiently and accurately. In the GB algorithm, the positions of atoms are determined iteratively and rely on other already determined positions of atoms, which cause the accumulation of numerical errors. The strategy of updating allows us to control the size of numerical errors. However, in the UBG algorithm updating requires implementing an expensive step that contributes up to *O(n^4^)* in the running time and the condition that the four base atoms to be updated must have all their distances known is quite strong. In this paper, the RUGB algorithm relaxes the condition for updating and incorporates the data structure for accessing neighbours of an atom. This results in an improvement of both the overall runtime and the numeric error over the UGB algorithm.

The RUGB algorithm has shown important properties of controlling numerical errors and effectiveness. However, this paper provides only theoretical studies of the method. The practical problems generally have distance ranges in a data set, such as NMR structure determination and protein structure prediction. In the future, we will address the application of RUGB methods in these cases. Also the theoretical results provide the upper bound of run-time when a sparse set of distances is given. More advanced methods should also be Applications of knowledge in graph theory or other advanced data structures may improve the algorithm further and will be a topic of future research.

## Competing interests

The authors declare that they have no competing interests.

## Authors' contributions

RTD carried out the programming and numerical tests. CE participated in the design of the study and helped to draft the manuscript. DW participated in the design of the study and helped to draft the manuscript. All authors read and approved the final manuscript.
